# Gynecological health: A missing link in comprehensive treatment monitoring for multiple sclerosis

**DOI:** 10.1177/13524585251346371

**Published:** 2025-06-18

**Authors:** Melika Arab Bafrani, Viviana Rios, Min Ji Kim, Ayushi Balan, Riley Bove

**Affiliations:** UCSF Weill Institute for Neurosciences, University of California San Francisco, San Francisco, CA, USA; UCSF Weill Institute for Neurosciences, University of California San Francisco, San Francisco, CA, USA; UCSF Weill Institute for Neurosciences, University of California San Francisco, San Francisco, CA, USA; UCSF Weill Institute for Neurosciences, University of California San Francisco, San Francisco, CA, USA; UCSF Weill Institute for Neurosciences, University of California San Francisco, San Francisco, CA, USA

**Keywords:** Vaginal complications, gynecological health, cervical cancer, HPV, immune suppression

## Abstract

Safety monitoring of disease-modifying therapies (DMTs) used to treat multiple sclerosis (MS) has largely overlooked the domain of gynecological health. This topical review aims to provide MS clinicians with an overview of the three categories of complications described to date, as well as risk mitigation strategies. These are increased risk of human papilloma virus (HPV) positivity and related cervical dysplasia/cancers; inflammatory and infectious vaginitis and susceptibility to bacterial vaginosis (BV); and herpesvirus infections, including genital Herpes Simplex Virus (HSV). Current knowledge may be biased due to limited studies and lack of gynecological focus in neurological encounters. Risk mitigation strategies include promoting HPV vaccination, following guidance for immune compromised individuals relating to cervical cancer screening and antiviral suppression, and proactive communication with patients about gynecological health when starting DMTs. Together, these might improve gynecological health and thereby quality of life in females with neuroinflammatory diseases.

## Highlights

- There is an unmet need to understand the effects of MS disease-modifying therapies (DMTs) on gynecological health
- DMTs have been associated with HPV-related cervical pathology, vaginitis, and herpesvirus infections
- Risk mitigation strategies could improve gynecological health and overall DMT safety profiles

## Introduction

Disease-modifying therapies (DMTs) are highly effective in preventing neuroinflammatory attacks that arise in multiple sclerosis (MS) and thereby, axonal loss and clinical progression. However, with prolonged use, complications can arise including lymphopenia, hypogammaglobinemia, infections, and colitis.^1^ One arena that remains inadequately explored is the potential impact of long-term use of DMTs on female gynecological health.^2^ Since reproductive and vaginal health are important components of overall well-being, understanding the potential impact of MS DMTs on gynecological complications is critical. Indeed, gynecologic cancer and cancer screening are identified as unmet needs in MS not only by clinicians but by patients.^3^

The goal of this topical review is to describe what is known about DMTs and gynecological health in women with MS. This is becoming more important with the widespread use of B-cell depleting therapies (BCDTs), with clinical trials and real-world studies demonstrating effective reduction of relapse risk and favorable safety and tolerability.^4^ Recent data point to potential elevated risk of vaginal inflammation, gynecological infections,^5^ and gynecological dysplasia and cancer.^6^ One possible mediating factor for these associations is the impact of DMTs with immunosuppressive properties on vaginal mucosal immunity, resulting in vaginal dysbiosis (VD, i.e. imbalances in microbial communities, which are more commonly studied in the gut) and infections.^7^ This review further emphasizes integrating such considerations into comprehensive MS management, optimizing treatment strategies to balance therapeutic benefits with reduction of gynecological risks. A roadmap for future research is proposed, with the goals of ultimately enhancing patient outcomes.

## Methods

This topical review sought to provide an in-depth review of the current understanding of the effects of DMTs on the gynecological and sexual health of women with MS. A search was conducted between 1 November 2024 and 1 February 2025 using three scientific databases (PubMed, Scopus, and Google Scholar) to gather relevant data and evidence. Articles were selected based on their relevance to the effects of DMTs on the gynecological health of women with MS. Keywords for the search included terms such as “disease-modifying therapy,” “multiple sclerosis,” “gynecological health,” “vaginal inflammation,” “immunosuppressive treatments,” “vaginal dysbiosis,” and “gynecological infections,” “HPV,” and “cervical cancer.” No restrictions were placed on country or article type. Studies with clinical trials, reviews, or case reports based on reliable scientific evidence were prioritized. A full list of the literature reviewed is presented in Supplementary Appendix 1.

## Results

### Immune regulation and the vaginal mucosa: overview

#### Physical and immunological barriers in vagina

The first-line physical defense for the female reproductive tract is an ectocervix and vagina lined with stratified non-keratinized squamous epithelium, which provides a protective barrier, further supported by chemical and biological barriers. An intact mucosa contains immune factors including cytokines, complement, antibodies, and antimicrobial peptides.^8^ Immune cells within the vaginal mucosa provide layers of innate (e.g. macrophages, natural killer (NK) cells, dendritic cells) and adaptive (CD4+ helper T cells, CD8+ cytotoxic T cells and CD19 B cells) immunity.^6^ B cells help maintain mucosal immunity by producing antibodies such as secretory IgA (sIgA) and IgM to protect against microbial colonization and inflammation, as well as IL-10 to regulate immune responses.^9^ Dysfunction in any component of this system is associated with heightened risk of herpes virus infections and cervical cancer linked to human papillomavirus (HPV).^10^

#### Vaginal microbiome

##### Role in immunity

Overall, maintaining a minimally diverse, lactobacilli-dominated vaginal bacterial microbiome (VBM) appears essential to promoting a healthy vaginal ecosystem and supporting host-microbial interactions through production of bacteriocins, hydrogen peroxide, and lactic acid.^11^ VD is characterized by a reduction in protective Lactobacillus species and greater microbial diversity. The presence of BV-associated species can raise vaginal pH, reduce antimicrobial defenses, and promote pathogenic growth, leading to BV, Chlamydia trachomatis, and vulvovaginal candidiasis.^12^ Changes in the VBM have been linked to gynecological sequelae including susceptibility to trichomoniasis, HPV, and genital herpes simplex virus-2 (HSV-2).^13^

#### Factors influencing the vaginal microbiome

Numerous factors influence VBM composition, emphasizing the complex interplay between behavioral, environmental, and physiological factors in maintaining vaginal health and preventing dysbiosis. These include age, reproductive stage, exogenous estrogens, local exposures (hygiene or sexual practices), and smoking. Broad-spectrum antibiotics like metronidazole can disrupt the vaginal microbiome by depleting Lactobacillus, allowing opportunistic microbes to thrive. Finally, immune-modulating therapies, such as DMTs for MS, can have variable effects on local mucosal tolerance mechanisms. potentially exacerbating underlying inflammatory disorders or other complications within the female reproductive tract.^14^

### MS DMTs, immune regulation, and gynecological sequelae

DMTs target the immune system through varied mechanisms of action, and their impact on T cells, B cells, and NK cells/subsets varies considerably, therefore, they could over time influence immune cells relevant to vaginal and mucosal health in different ways. For example, BCDTs could over time by lower secretory IgA and disrupt vaginal microbiota.^15^ Sphingosine-1-receptor (S1P) modulators impair lymphocyte trafficking, leading to systemic immunosuppression that could reduce vaginal immune surveillance and compromise microbiome stability.^16^ Natalizumab, by inhibiting α4-integrin, prevents immune cell migration, potentially altering mucosal homeostasis and microbial diversity.^17^ Overall, further research is needed to clarify potential mechanisms underlying DMT-induced vaginal microbiome shifts and their clinical implications.

#### Specific gynecological outcomes

##### Cervical cancer and dysplasia

HPV enters the basal keratinocytes through micro-abrasions, replicating during epithelial migration, and releases virions at the surface, evading systemic immunity. This implies that any effects of DMTs on HPV clearance would be mediated by local effects on immune surveillance,^18^ thereby decreasing HPV clearance and increasing the likelihood of cervical dysplasia and cancer. Recent studies have raised concerns regarding the incidence of refractory HPV infections in patients treated with DMTs,^19,20^ and this topic is particularly important given that researchers and patients alike identify gynecological cancers as a research gap in MS.^3^

To contextualize a potential link between DMTs and cervical cancer, it is important to consider the timescale as well as potential confounders. Cervical carcinogenesis is a prolonged process, and the 2- to 4-year follow-up in typical MS cohorts may be insufficient to fully capture the long-term oncogenic risks of high-efficacy immunotherapies. Furthermore, the risk of cervical cancer in women with MS could be influenced by diagnostic neglect, whereby more disabled women are underrepresented in cancer prevention programs, and may be less likely to receive recommended cancer screening.^21,22^ Findings from a nationwide cohort study, of no MS-associated risk of female genital organ cancer until after 1996, suggests this could be due to the introduction of DMTs.^23^ Regarding specific DMTs, to date, the data are mixed. This is the case overall for associations between natalizumab and cervical abnormalities^24,25^ with larger studies, including a Swedish registry and the AFFIRM trial, reporting no significant increased risk of cervical cancer; one case of cervical carcinoma in situ was reported in AFFIRM.^26^ Glatiramer acetate and interferon-beta have not been linked to cervical cancer risk in MS,^27^ while fingolimod and cladribine have been linked to increased cervical abnormalities.^6^

An Australian multicenter cohort study (1998–2019) demonstrated higher incidence of abnormal cervical screening tests (CSTs), including both low- and high-grade squamous intraepithelial lesions (LSIL-HSIL), among women with MS exposed to moderate-high efficacy DMTs (in this context, fingolimod, cladribine, dimethyl fumarate, natalizumab, alemtuzumab and BCDTs), relative to non-exposed individuals.^28^ This pattern aligns with other immunocompromised populations (HIV, solid organ transplant recipients), where persistent HPV infections heighten the risk of cervical pre-cancer and progression to malignancy.^29,30^ Despite these concerns, current evidence regarding the relationship between BCDT exposure in MS and cervical abnormalities remains inconclusive. A Swedish cohort study evaluating the incidence of cervical intraepithelial neoplasia grade 3 (CIN3) in individuals receiving natalizumab, fingolimod, and rituximab, identified a borderline significant elevation in risk for fingolimod (HR: 1.63, 95% CI: 0.94–2.82), but not the other DMTs.^26^

Ongoing post-marketing studies, such as the Verismo study evaluating malignancy incidence in ocrelizumab-treated patients, are critical for understanding the long-term safety profile of DMTs. The findings from these studies may inform future patient care strategies, including enhanced cancer screening protocols and individualized risk-based treatment decisions.^31^

Further longitudinal investigations with extended follow-up periods are warranted to elucidate whether BCDT use in MS predisposes patients to an HPV-associated cervical abnormalities and malignancies.

#### Risk mitigation strategies

An intentional and comprehensive approach is required to reduce the risk of HPV activation and cervical dysplasia/cancer in women with MS on DMTs that alter immune function and reduce surveillance against HPV, particularly those affecting lymphocyte presence and function, and with longer-term use of DMTs.^32^ To achieve this, collaboration between neurologists, gynecologists, and primary care providers is key to educate patients about risks of HPV-related complications and encourage vaccines prior to DMT initiation.^33^ The current Centers for Disease Control and Prevention (CDC) guidelines recommend three doses of HPV vaccination for individuals up to age 26 and ages 27–45 if deemed beneficial, noting that many eligible individuals may have missed the initial 2006 vaccination rollout.^34^ Improving vaccination rates could ultimately reduce HPV-related disease burden in this at-risk demographic. Immunocompromised women are expected to benefit from vaccination, even if they have previously been exposed to the virus, as the vaccine-induced antibodies can prevent new infections, reinfections with eliminated HPV types, and the spread or reactivation of the virus within the genital tract.^35^

**Figure 1.**
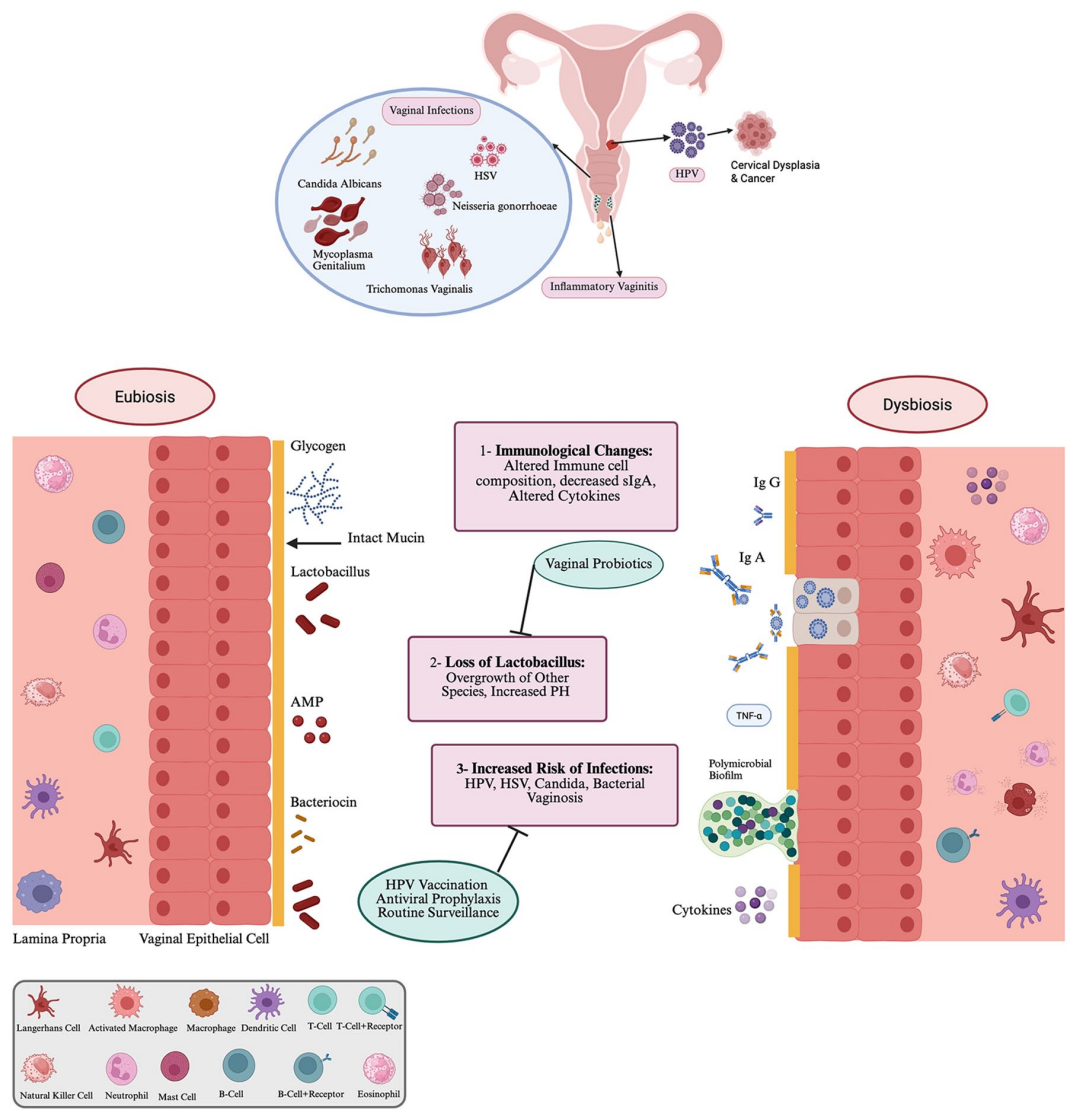
Overview of gynecological complications associated with Disease-Modifying Therapies for Multiple Sclerosis. Panel A. Three major categories of complications are (1) HPV infection and cervical dysplasia. (2) Infectious and inflammatory vaginitis and (3) HSV infection. Panel B. Overview of putative mechanisms leading to vaginal dysbiosis, and its sequelae. These figures were created using BioRender.com and is included with permission under a BioRender Publication License.

Incorporating routine cervical cancer screenings, like Pap smears, into standard care alongside vaccination is crucial for early detection of HPV-related abnormalities. Due to the heightened likelihood of abnormal Pap smear results progressing to precancerous or cancerous conditions, follow-up colposcopy can further evaluate abnormal screening findings.^36^ Guidelines vary by country and society; but more frequent HPV, cytological, or co-testing is recommended by the American Society for Colposcopy and Cervical Pathology in individuals with immunosuppression.^33^ Proactive counseling, vaccination and increased screening for atypia could substantially reduce the risk of cervical cancer associated with immune suppression (Figure 1).^33^

#### Inflammatory vaginitis and bacterial vaginosis

A growing body of work is linking the use of MS DMTs with a variety of vaginal inflammatory and infectious disorders, particularly implicating B-cell depletion. Unfortunately, the absence of systematic data collection limits accurate risk assessment, making current findings subject to bias.

#### Inflammatory vaginitis

*Vulvovaginal pyoderma gangrenosum (PG)* presents as painful vulvar or perianal ulcerations, with biopsies typically revealing neutrophilic dermatoses thought to result from an abnormal immune response or disrupted neutrophil chemotaxis. PG has been reported in women with hematological and rheumatologically conditions receiving rituximab,^37,38^ and some severe cases of PG associated with B-cell depletion are documented in women with MS, as well.^39,40^

*Desquamative inflammatory vaginitis (DIV)* is more common in women with autoimmune disorders and those receiving specific immunomodulatory therapies. There are some case reports of severe and recurrent DIV following treatment with rituximab, ocrelizumab, and natalizumab in MS.^41–43^ Another study noted no significant difference in inflammatory vaginitis rates between women with MS on BCDTs vs other DMTs, but vaginitis was common in both groups.^44^ At present, it is not possible to distinguish whether these findings reflect treatment effects or an autoinflammatory process, given the higher autoimmunity risk in MS patients. The clinical impact of inflammatory vaginitis (IV) requires consideration. Indeed, it can have detrimental effects on quality of life and reproductive health, as detailed in a case series of three women with MS receiving BCDT, all of whom changed DMTs due to persistent and refractory IV symptoms.^41^

### Infectious vaginitis

A real-world study of women with MS reported an elevated risk of yeast vaginitis and bacterial vaginosis (BV) compared to the general population. In addition, rituximab use was associated with an increased risk of these conditions.^7^ The available data are hindered by small sample sizes, confounding factors, and underdiagnosis and mismanagement.

#### Risk mitigation strategies

A proactive approach is required to manage the potential risk and sequelae of inflammatory and infectious vaginitis in patients receiving DMTs. This is especially important because patients may not associate vaginal symptoms with their MS management, and neurologists may not routinely enquire about patients’ gynecological health. Prior to initiating DMTs, counseling should address potential effects on vaginal health, empowering patients to recognize symptoms and seek medical attention when necessary. Clinicians should remain vigilant for signs of vaginal infections in MS patients receiving DMTs, incorporating discussions on vaginal health into safety monitoring. Symptoms like abnormal discharge, discomfort, or pruritus should prompt early consult with the patient’s primary care clinician or gynecologist to minimize diagnostic delays and consider interventions targeting the alleviation of dysbiosis and associated symptoms. Treatments may include antibiotics (CDC,)^45^ and when appropriate, antifungal treatments, *Lactobacillus* probiotics, topical steroids, or intravaginal hormonal therapies for hormonal imbalances or atrophic vaginitis.^46^ Managing additional risk factors like smoking and stress should also be considered to support vaginal health.

#### Viral infections

Most MS therapies are associated with an elevated risk of viral infections, including herpes simplex virus (HSV) reactivation.^47^ Alemtuzumab and fingolimod, in particular, have been associated with markedly elevated rates of HSV infections, some of which were severe enough to require hospitalization.^48,49^ Ocrelizumab appears to increase the risk of HSV, although most cases were mild to moderate and none resulted in long-term complications.^50^ Other DMTs for MS, such as teriflunomide, dimethyl fumarate, and mitoxantrone, do not show a clear association with an increased frequency or severity of HSV.

#### Risk mitigation strategies

Given the potential for DMTs used in MS to increase the risk of HSV infections, it is important to implement strong preventive strategies to reduce the risk of viral reactivation and associated complications. CDC-recommended strategies may include screening for prior viral exposure, and use of long-term low-dose preventive antivirals such as acyclovir, valacyclovir, and famciclovir in individuals who have experienced recurrence^51^—with specific dose and duration of antiviral prophylaxis tailored based on clinical context and individual risk factors. For patients on DMTs who experience viral reactivation, early recognition, prompt initiation of antiviral therapy, and vigilant monitoring are essential for controlling outbreaks and preventing severe complications and to ensure timely intervention and optimize patient recovery.

## Discussion

The significant advances in MS treatment in recent years have been accompanied by emerging concerns about how DMTs might alter key regulatory mechanisms of gynecological health: innate immunity, the microbiome, and/or mucosal histology. This topical review identified three areas of concern: HPV activation and cervical dysplasia/cancer, inflammatory and infectious vaginitis, and HSV infections (Figure 1). Overall, studies were sparse, and some were likely affected by methodological limitations or bias. Furthermore, mechanistic investigations are lacking, particularly on the effect of DMTs on the vaginal microbiome. This scientific gap is particularly important given that the vaginal and gut microbiome represents key dimensions of materno-fetal immunoregulation,^52^ alongside placental and breastmilk transfer of immunoglobulins.^53^ Therefore, future studies should evaluate how maternal immune status might influence the development of microbial reservoirs and immune function in newborns.

From a research standpoint, incorporating routine gynecological assessments and microbiome analyses in MS clinical trials could provide valuable insights into the long-term impact of immunomodulatory therapies on reproductive health. Real-world studies could evaluate the role of DMT switch after symptoms, and the prophylactic role of vaginal probiotics, on gynecological outcomes. From a clinical practice standpoint, monitoring (e.g. through a brief gynecological health screen) and addressing potential gynecological impacts of DMTs could minimize risks of their use. Approaches are summarized in Table 1. Overall, clinicians should recognize gynecological complications as potential sequelae of long-term immune suppression, and adopt a multidisciplinary approach that integrates gynecological counseling and screening as integral to the MS therapeutic strategy. By doing so, they could mitigate gynecological risks, optimize patient outcomes, and enhance overall well-being.

**Table 1.** Overview of gynecological complications associated with disease-modifying therapies used for the treatment of multiple sclerosis.

	HPV and cervical dysplasia	Inflammatory and infectious vaginitis	HSV infections
Clinical syndrome	Increased risk of cervical dysplasia and cancer in MS patients on immunosuppressive DMT	Higher prevalence of bacterial vaginosis (BV) and candidiasis in MS patients	Increased HSV reactivation due to immunosuppression
Pathophysiology	Immune dysregulation leads to reduced viral clearance^10^	Altered vaginal microbiome, immune suppression affecting mucosal defense, loss of regulatory B cell function influencing cytokines^41^	T-cell dysfunction and reduced antiviral response due to DMTs^47^
Case reports in MS	Reports of greater HPV persistence and rapid progression of cervical dysplasia to cancer in women with MS on DMTs^24,25^	Reports of recurrent and refractory vaginitis in women with MS, particularly those on S1P receptor modulators^44^ and BCDTs^41^	Cases of severe or recurrent HSV outbreaks in patients with MS receiving DMTs^50^
Prevention and treatment strategies	3-dose HPV vaccination for individuals up to age 26, and ages 27–45 if beneficial^36^	Probiotic use, antifungal/antibacterial treatments, personalized hygiene recommendations	Antiviral prophylaxis in high-risk patients, monitoring during DMT therapy^51^
Guidance in immune-compromised individuals	HPV screening before starting DMTs, Individualized risk assessment More frequent HPV, cytological testing for cervical dysplasia, or co-testing, and colposcopy for any abnormal cervical screening tests^34^	Consideration of switch from anti-CD20 therapy to alternative DMTs in those with recurrent infection/inflammatory vaginitis^41^	Suppressive antiviral therapy (acyclovir, famciclovir or valacyclovir)^54^ tailored to the clinical context.

BCDT: B-cell depleting therapy. BV: bacterial vaginosis. DMT: disease-modifying therapy. HPV: human papilloma virus. HSV: herpes simplex virus.

## Supplemental Material

sj-docx-1-msj-10.1177_13524585251346371 – Supplemental material for Gynecological health: A missing link in comprehensive treatment monitoring for multiple sclerosis Supplemental material, sj-docx-1-msj-10.1177_13524585251346371 for Gynecological health: A missing link in comprehensive treatment monitoring for multiple sclerosis by Melika Arab Bafrani, Viviana Rios, Min Ji Kim, Ayushi Balan and Riley Bove in Multiple Sclerosis Journal

## References

[1] Leung MWY Garde E Uitdehaag BMJ , et al. The relative risk of infection in people with multiple sclerosis using disease-modifying treatment: A systematic review of observational studies. Neurol Sci 2025; 46: 2555–2569. 39920457 10.1007/s10072-025-08018-9 PMC12084263

[2] Athar F Karmani M Templeman NM . Metabolic hormones are integral regulators of female reproductive health and function. Biosci Rep 2024; 44: BSR20231916. 10.1042/BSR20231916 PMC10830447 38131197

[3] Ross L Finlayson M Amato MP , et al. Priority setting: Women’s health topics in multiple sclerosis. Front Neurol 2024; 15: 1355817. 38440114 10.3389/fneur.2024.1355817 PMC10910071

[4] Comi G Bar-Or A Lassmann H , et al. Role of B cells in multiple sclerosis and related disorders. Ann Neurol 2021; 89: 13–23. 33091175 10.1002/ana.25927 PMC8007167

[5] Filikci Z Jensen RM Thorup Sellebjerg F . Inflammatory vaginitis associated with long-term rituximab treatment in a patient with multiple sclerosis. BMJ Case Rep 2022; 15: e250425. 10.1136/bcr-2022-250425 PMC9685188 36414352

[6] Bridge F Brotherton JML Foong Y , et al. Risk of cervical pre-cancer and cancer in women with multiple sclerosis exposed to high efficacy disease modifying therapies. Front Neurol 2023; 14: 1119660. 36846149 10.3389/fneur.2023.1119660 PMC9950275

[7] Langer-Gould AM Smith JB Gonzales EG , et al. Multiple sclerosis, disease-modifying therapies, and infections. Neurol Neuroimmunol Neuroinflamm 2023; 10: e200164. 10.1212/NXI.0000000000200164 PMC10574822 37813594

[8] Monin L Whettlock EM Male V . Immune responses in the human female reproductive tract. Immunology 2020; 160: 106–115. 31630394 10.1111/imm.13136 PMC7218661

[9] Chen K Magri G Grasset EK , et al. Rethinking mucosal antibody responses: IgM, IgG and IgD join IgA. Nat Rev Immunol 2020; 20: 427–441. 32015473 10.1038/s41577-019-0261-1 PMC10262260

[10] Sausen DG Shechter O Gallo ES , et al. Herpes simplex virus, human papillomavirus, and cervical cancer: Overview, relationship, and treatment implications. Cancers 2023; 15: 3692. 37509353 10.3390/cancers15143692 PMC10378257

[11] De Seta F Campisciano G Zanotta N , et al. The vaginal community state types microbiome-immune network as key factor for bacterial vaginosis and aerobic vaginitis. Front Microbiol 2019; 10: 2451. 31736898 10.3389/fmicb.2019.02451 PMC6831638

[12] Han Y Liu Z Chen T . Role of vaginal microbiota dysbiosis in gynecological diseases and the potential interventions. Front Microbiol 2021; 12: 643422. 34220737 10.3389/fmicb.2021.643422 PMC8249587

[13] Gupta S Kakkar V Bhushan I . Crosstalk between vaginal microbiome and female health: A review. Microbial Pathogenesis 2019; 136: 103696. 31449855 10.1016/j.micpath.2019.103696

[14] Baecher -Allan C Kaskow BJ Weiner HL . Multiple sclerosis: Mechanisms and immunotherapy. Neuron 2018; 97: 742–768. 29470968 10.1016/j.neuron.2018.01.021

[15] Canto-Gomes J Boleixa D Teixeira C , et al. Distinct disease-modifying therapies are associated with different blood immune cell profiles in people with relapsing-remitting multiple sclerosis. Int Immunopharmacol 2024; 131: 111826. 38461632 10.1016/j.intimp.2024.111826

[16] Harris S Feagan BG Hanauer S , et al. Ozanimod differentially impacts circulating lymphocyte subsets in patients with moderately to severely active Crohn’s disease. Dig Dis Sci 2024; 69: 2044–2054. 38568396 10.1007/s10620-024-08391-z PMC11162376

[17] Pang X He X Qiu Z , et al. Targeting integrin pathways: Mechanisms and advances in therapy. Sig Trans Target Therap 2023; 8: 1. 10.1038/s41392-022-01259-6 PMC9805914 36588107

[18] Zhou C Tuong ZK Frazer IH . Papillomavirus immune evasion strategies target the infected cell and the local immune system. Front Oncol 2019; 9: 682. 31428574 10.3389/fonc.2019.00682 PMC6688195

[19] Paybast S Ashtari F Moghaddam NB , et al. Investigating treatment alternatives for fingolimod in patients with multiple sclerosis developed refractory fingolimod-related genital Human Papilloma Virus (HPV) infection. Mult Scler Relat Disord 2025; 95: 106284. 39908723 10.1016/j.msard.2025.106284

[20] Mhanna E Nouchi A Louapre C , et al. Human papillomavirus lesions in 16 MS patients treated with fingolimod: Outcomes and vaccination. Mult Scler J 2021; 27: 1794–1798. 10.1177/1352458521991433 33629615

[21] Iezzoni LI Kurtz SG Rao SR . Trends in pap testing over time for women with and without chronic disability. Am J Prev Med 2016; 50: 210–219. 26372417 10.1016/j.amepre.2015.06.031

[22] Dobos K Healy B Houtchens M . Access to preventive health care in severely disabled women with multiple sclerosis. Int J MS Care 2015; 17: 200–205. 26300706 10.7224/1537-2073.2013-046 PMC4542715

[23] Grytten N Myhr KM Celius EG , et al. Incidence of cancer in multiple sclerosis before and after the treatment era: A registry- based cohort study. Mult Scler Relat Disord 2021; 55: 103209. 34419754 10.1016/j.msard.2021.103209

[24] Wan KM Oehler MK . Rapid progression of low-grade cervical dysplasia into invasive cancer during natalizumab treatment for relapsing remitting multiple sclerosis. Case Rep Oncol 2019; 12: 59–62. 31043942 10.1159/000496198 PMC6477472

[25] Durrieu G Dardonville Q Clanet M , et al. Cervical dysplasia in a patient with multiple sclerosis treated with natalizumab. Fundam Clin Pharmacol 2019; 33: 125–126. 29935014 10.1111/fcp.12394

[26] Alping P Askling J Burman J , et al. Cancer risk for fingolimod, natalizumab, and rituximab in multiple sclerosis patients. Ann Neurol 2020; 87: 688–699. 32056253 10.1002/ana.25701

[27] Doosti R Togha M Moghadasi AN , et al. Evaluation of the risk of cervical cancer in patients with Multiple Sclerosis treated with cytotoxic agents: A cohort study. Iran J Neurol 2018; 17: 64–70. 30210730 PMC6131331

[28] Bridge F Brotherton J Stankovich J , et al. Risk of cervical abnormalities for women with multiple sclerosis treated with moderate-efficacy and high-efficacy disease-modifying therapies. Neurology 2024; 102: e208059. 10.1212/WNL.0000000000208059 38306594

[29] Reusser NM Downing C Guidry J , et al. HPV Carcinomas in immunocompromised patients. J Clin Med 2015; 4: 260–281. 26239127 10.3390/jcm4020260 PMC4470124

[30] Grulich AE van Leeuwen MT Falster MO , et al. Incidence of cancers in people with HIV/AIDS compared with immunosuppressed transplant recipients: A meta-analysis. Lancet 2007; 370: 59–67. 17617273 10.1016/S0140-6736(07)61050-2

[31] Wormser D Evershed J Ferreira G , et al. VERISMO: A post-marketing safety study to determine the incidence of all malignancies and breast cancer in patients with multiple sclerosis treated with ocrelizumab (P4.2-043). Neurology 2019; 92: P42-043.

[32] Bayounis M Bin Mahfooz M Assiri M , et al. Risk of malignancy and the use of disease-modifying therapy in multiple sclerosis: Exploring the role of DMT in a multi-center study. Front Neurol 2024; 15: 1492678. 39734635 10.3389/fneur.2024.1492678 PMC11681499

[33] Moscicki AB Flowers L Huchko MJ , et al. Updated review for guidelines for cervical cancer screening in immunosuppressed women without HIV infection. J Low Genit Tract Dis 2025; 29: 168–179. 39804372 10.1097/LGT.0000000000000866 PMC11939099

[34] Markowitz LE Tsu V Deeks SL , et al. Human papillomavirus vaccine introduction: The first five years. Vaccine 2012; 30(Suppl. 5): F139–F148. 10.1016/j.vaccine.2012.05.039 23199957

[35] Clifford GM Gonçalves MA Franceschi S . Human papillomavirus types among women infected with HIV: A meta-analysis. AIDS 2006; 20: 2337–2344. 17117020 10.1097/01.aids.0000253361.63578.14

[36] Moscicki A-B Flowers L Huchko MJ , et al. Guidelines for cervical cancer screening in immunosuppressed women without HIV infection. J Lower Genit Tract Dis 2019; 23: 87–101. 10.1097/LGT.0000000000000468 30907775

[37] Sotzen JR Stratman EJ . Vulvovaginal pyoderma gangrenosum associated with rituximab use in 2 patients with rheumatoid arthritis. JAAD Case Reports 2021; 10: 75–77. 33778138 10.1016/j.jdcr.2021.02.013 PMC7985273

[38] Bell AP Jr Custer MK Presley S . Pyoderma gangrenosum in a 75-year-old male with follicular diffuse large B-cell lymphoma. Cureus 2024; 16: e67234. 10.7759/cureus.67234 PMC11335176 39165616

[39] Klumpp A Luessi F Engel S , et al. Ocrelizumab-induced vulvovaginal pyoderma gangrenosum in a patient with relapsing-remitting multiple sclerosis. JAAD Case Rep 2022; 28: 24–27. 36097621 10.1016/j.jdcr.2022.08.005 PMC9463556

[40] Parrotta E Kopinsky H Abate J , et al. It’s not always an infection: Pyoderma gangrenosum of the urogenital tract in two patients with multiple sclerosis treated with rituximab. Mult Scler Relat Disord 2023; 70: 104483. 36580875 10.1016/j.msard.2022.104483

[41] Levine L Son J Yu A , et al. Inflammatory vaginitis in four B-cell suppressed women with multiple sclerosis. Mult Scler Relat Disord 2024; 82: 105387. 38134606 10.1016/j.msard.2023.105387

[42] Makris GM Mene J Fotiou A , et al. Gynecological adverse effects of natalizumab administration: Case report and review of the literature. Mult Scler Relat Disord 2018; 25: 46–49. 30032043 10.1016/j.msard.2018.07.024

[43] Longbrake EE Cross AH . Effect of multiple sclerosis disease-modifying therapies on B cells and humoral immunity. JAMA Neurol 2016; 73: 219–225. 26720195 10.1001/jamaneurol.2015.3977

[44] Conway S Dodson C Hui G , et al. Inflammatory vaginitis in women with multiple sclerosis: A retrospective analysis of B-cell depleting therapy compared to other disease modifying therapies. Mult Scler Relat Disord 2024; 92: 105921. 39454471 10.1016/j.msard.2024.105921

[45] Centers for Disease Control and Prevention. Bacterial vaginosis – 2021 STD treatment guidelines. CDC, https://www.cdc.gov/std/treatment-guidelines/bv.htm (2021, accessed 21 May 2025).

[46] Mitchell CM . Assessment and treatment of vaginitis. Obstet Gynecol 2024; 144: 765–781. 38991218 10.1097/AOG.0000000000005673

[47] Epstein DJ Dunn J Deresinski S . Infectious complications of multiple sclerosis therapies: Implications for screening, prophylaxis, and management. Open Forum Infect Dis 2018; 5: ofy174. 10.1093/ofid/ofy174 PMC6080056 30094293

[48] Coles AJ Twyman CL Arnold DL , et al. Alemtuzumab for patients with relapsing multiple sclerosis after disease-modifying therapy: A randomised controlled phase 3 trial. Lancet 2012; 380: 1829–1839. 23122650 10.1016/S0140-6736(12)61768-1

[49] Arvin AM Wolinsky JS Kappos L , et al. Varicella-zoster virus infections in patients treated with fingolimod: Risk assessment and consensus recommendations for management. JAMA Neurology 2015; 72: 31–39. 25419615 10.1001/jamaneurol.2014.3065 PMC5391035

[50] Hauser SL Bar-Or A Comi G , et al. Ocrelizumab versus interferon beta-1a in relapsing multiple sclerosis. N Engl J Med 2017; 376: 221–234. 28002679 10.1056/NEJMoa1601277

[51] Centers for Disease Control and Prevention. Genital herpes: CDC treatment guidelines 2025. Atlanta (GA): CDC, https://www.cdc.gov/std/treatment-guidelines/herpes.htm (2025, accessed 21 May 2021).

[52] McCauley KE Rackaityte E LaMere B , et al. Heritable vaginal bacteria influence immune tolerance and relate to early-life markers of allergic sensitization in infancy. Cell Rep Med 2022; 3: 100713. 35932762 10.1016/j.xcrm.2022.100713 PMC9418802

[53] Sereme Y Toumi E Saifi E , et al. Maternal immune factors involved in the prevention or facilitation of neonatal bacterial infections. Cell Immunol 2024; 395–396: 104796. 10.1016/j.cellimm.2023.104796 38104514

[54] Centers for Disease Control and Prevention. 2021 STD treatment guidelines: Genital herpes simplex virus infection. Atlanta (GA): CDC, https://www.cdc.gov/std/treatment-guidelines/herpes.htm (2021, accessed 21 May 2025).

