# Supplementary material for: Gynecological health: A missing link in comprehensive treatment monitoring for multiple sclerosis
Source: Mult Scler. 2025 Jun 18;31(9):1023–31. doi: 10.1177/13524585251346371 (PMC12357974; doi:10.1177/13524585251346371)
Supplement: sj-docx-1-msj-10.1177_13524585251346371 – Supplemental material for Gynecological health: A missing link in comprehensive treatment monitoring for multiple sclerosis [file sj-docx-1-msj-10.1177_13524585251346371.docx]

**REFERENCES**

1. Dempsey JP, Wu L, Balshi A, Jun C, Baber U, Sloane JA. Worsening of lymphopenia in patients with multiple sclerosis when switched from dimethyl fumarate to diroximel fumarate. Mult Scler Relat Disord. 2024;89:105737.

2. Baeva ME, Metz LM, Greenfield J, Camara-Lemarroy CR. Simple parameters from complete blood count predict lymphopenia, adverse effects and efficacy in people with MS treated with dimethyl fumarate. Mult Scler Relat Disord. 2023;74:104699.

3. Fischer S, Proschmann U, Akgün K, Ziemssen T. Lymphocyte Counts and Multiple Sclerosis Therapeutics: Between Mechanisms of Action and Treatment-Limiting Side Effects. Cells. 2021;10(11).

4. Krajnc N, Bsteh G, Berger T, Mares J, Hartung HP. Monoclonal Antibodies in the Treatment of Relapsing Multiple Sclerosis: an Overview with Emphasis on Pregnancy, Vaccination, and Risk Management. Neurotherapeutics. 2022;19(3):753-73.

5. Wingerchuk DM, Carter JL. Multiple Sclerosis: Current and Emerging Disease-Modifying Therapies and Treatment Strategies. Mayo Clinic Proceedings. 2014;89(2):225-40.

6. Leung MWY, Garde E, Uitdehaag BMJ, Klungel OH, Bazelier MT. The relative risk of infection in people with multiple sclerosis using disease-modifying treatment: a systematic review of observational studies. Neurol Sci. 2025.

7. Mallepally N, Abu-Sbeih H, Ahmed O, Chen E, Shafi MA, Neelapu SS, et al. Clinical Features of Rituximab-associated Gastrointestinal Toxicities. Am J Clin Oncol. 2019;42(6):539-45.

8. Lamaita R, Melo C, Laranjeira C, Barquero P, Gomes J, Silva-Filho A. Multiple Sclerosis in Pregnancy and its Role in Female Fertility: A Systematic Review. JBRA Assist Reprod. 2021;25(3):493-9.

9. Athar F, Karmani M, Templeman NM. Metabolic hormones are integral regulators of female reproductive health and function. Biosci Rep. 2024;44(1).

10. Comi G, Bar-Or A, Lassmann H, Uccelli A, Hartung HP, Montalban X, et al. Role of B Cells in Multiple Sclerosis and Related Disorders. Ann Neurol. 2021;89(1):13-23.

11. Filikci Z, Jensen RM, Thorup Sellebjerg F. Inflammatory vaginitis associated with long-term rituximab treatment in a patient with multiple sclerosis. BMJ Case Rep. 2022;15(11).

12. Bridge F, Brotherton JML, Foong Y, Butzkueven H, Jokubaitis VG, Van der Walt A. Risk of cervical pre-cancer and cancer in women with multiple sclerosis exposed to high efficacy disease modifying therapies. Front Neurol. 2023;14:1119660.

13. Langer-Gould AM, Smith JB, Gonzales EG, Piehl F, Li BH. Multiple Sclerosis, Disease-Modifying Therapies, and Infections. Neurol Neuroimmunol Neuroinflamm. 2023;10(6).

14. Monin L, Whettlock EM, Male V. Immune responses in the human female reproductive tract. Immunology. 2020;160(2):106-15.

15. Zhao C, Yan S, Song Y, Xia X. Roles of Antimicrobial Peptides in Gynecological Cancers. Int J Mol Sci. 2022;23(17).

16. Yarbrough VL, Winkle S, Herbst-Kralovetz MM. Antimicrobial peptides in the female reproductive tract: a critical component of the mucosal immune barrier with physiological and clinical implications. Human Reproduction Update. 2014;21(3):353-77.

17. Sausen DG, Shechter O, Gallo ES, Dahari H, Borenstein R. Herpes Simplex Virus, Human Papillomavirus, and Cervical Cancer: Overview, Relationship, and Treatment Implications. Cancers (Basel). 2023;15(14).

18. van Teijlingen NH, Eder J, Sarrami-Forooshani R, Zijlstra-Willems EM, Roovers JWR, van Leeuwen E, et al. Immune activation of vaginal human Langerhans cells increases susceptibility to HIV-1 infection. Sci Rep. 2023;13(1):3283.

19. Poston TB, Lee DE, Darville T, Zhong W, Dong L, O'Connell CM, et al. Cervical Cytokines Associated With Chlamydia trachomatis Susceptibility and Protection. J Infect Dis. 2019;220(2):330-9.

20. Chen K, Magri G, Grasset EK, Cerutti A. Rethinking mucosal antibody responses: IgM, IgG and IgD join IgA. Nat Rev Immunol. 2020;20(7):427-41.

21. De Seta F, Campisciano G, Zanotta N, Ricci G, Comar M. The Vaginal Community State Types Microbiome-Immune Network as Key Factor for Bacterial Vaginosis and Aerobic Vaginitis. Front Microbiol. 2019;10:2451.

22. Chen X, Lu Y, Chen T, Li R. The Female Vaginal Microbiome in Health and Bacterial Vaginosis. Frontiers in Cellular and Infection Microbiology. 2021;11.

23. Chee WJY, Chew SY, Than LTL. Vaginal microbiota and the potential of Lactobacillus derivatives in maintaining vaginal health. Microbial Cell Factories. 2020;19(1):203.

24. Hooks KB, O'Malley MA. Dysbiosis and Its Discontents. mBio. 2017;8(5).

25. Winter SE, Bäumler AJ. Gut dysbiosis: Ecological causes and causative effects on human disease. Proc Natl Acad Sci U S A. 2023;120(50):e2316579120.

26. Alcendor DJ. Evaluation of Health Disparity in Bacterial Vaginosis and the Implications for HIV-1 Acquisition in African American Women. Am J Reprod Immunol. 2016;76(2):99-107.

27. Chen X, Lu Y, Chen T, Li R. The Female Vaginal Microbiome in Health and Bacterial Vaginosis. Front Cell Infect Microbiol. 2021;11:631972.

28. Baldewijns S, Sillen M, Palmans I, Vandecruys P, Van Dijck P, Demuyser L. The Role of Fatty Acid Metabolites in Vaginal Health and Disease: Application to Candidiasis. Front Microbiol. 2021;12:705779.

29. Chee WJY, Chew SY, Than LTL. Vaginal microbiota and the potential of Lactobacillus derivatives in maintaining vaginal health. Microb Cell Fact. 2020;19(1):203.

30. Saadaoui M, Singh P, Ortashi O, Al Khodor S. Role of the vaginal microbiome in miscarriage: exploring the relationship. Front Cell Infect Microbiol. 2023;13:1232825.

31. Gupta S, Kakkar V, Bhushan I. Crosstalk between Vaginal Microbiome and Female Health: A review. Microbial Pathogenesis. 2019;136:103696.

32. Mitra A, MacIntyre DA, Marchesi JR, Lee YS, Bennett PR, Kyrgiou M. The vaginal microbiota, human papillomavirus infection and cervical intraepithelial neoplasia: what do we know and where are we going next? Microbiome. 2016;4(1):58.

33. van de Wijgert J. The vaginal microbiome and sexually transmitted infections are interlinked: Consequences for treatment and prevention. PLoS Med. 2017;14(12):e1002478.

34. Ziklo N, Vidgen ME, Taing K, Huston WM, Timms P. Dysbiosis of the Vaginal Microbiota and Higher Vaginal Kynurenine/Tryptophan Ratio Reveals an Association with Chlamydia trachomatis Genital Infections. Front Cell Infect Microbiol. 2018;8:1.

35. Shannon B, Gajer P, Yi TJ, Ma B, Humphrys MS, Thomas-Pavanel J, et al. Distinct Effects of the Cervicovaginal Microbiota and Herpes Simplex Type 2 Infection on Female Genital Tract Immunology. J Infect Dis. 2017;215(9):1366-75.

36. Ceccarani C, Foschi C, Parolin C, D'Antuono A, Gaspari V, Consolandi C, et al. Diversity of vaginal microbiome and metabolome during genital infections. Sci Rep. 2019;9(1):14095.

37. Ma B, Forney LJ, Ravel J. Vaginal microbiome: rethinking health and disease. Annu Rev Microbiol. 2012;66:371-89.

38. Zapata HJ, Quagliarello VJ. The microbiota and microbiome in aging: potential implications in health and age-related diseases. J Am Geriatr Soc. 2015;63(4):776-81.

39. Plummer EL, Vodstrcil LA, Fairley CK, Tabrizi SN, Garland SM, Law MG, et al. Sexual practices have a significant impact on the vaginal microbiota of women who have sex with women. Sci Rep. 2019;9(1):19749.

40. Mulder M, Radjabzadeh D, Hassing RJ, Heeringa J, Uitterlinden AG, Kraaij R, et al. The effect of antimicrobial drug use on the composition of the genitourinary microbiota in an elderly population. BMC Microbiology. 2019;19(1):9.

41. Hickey RJ, Zhou X, Settles ML, Erb J, Malone K, Hansmann MA, et al. Vaginal microbiota of adolescent girls prior to the onset of menarche resemble those of reproductive-age women. mBio. 2015;6(2).

42. Kaur H, Merchant M, Haque MM, Mande SS. Crosstalk Between Female Gonadal Hormones and Vaginal Microbiota Across Various Phases of Women's Gynecological Lifecycle. Front Microbiol. 2020;11:551.

43. Nunn KL, Ridenhour BJ, Chester EM, Vitzthum VJ, Fortenberry JD, Forney LJ. Vaginal Glycogen, Not Estradiol, Is Associated With Vaginal Bacterial Community Composition in Black Adolescent Women. J Adolesc Health. 2019;65(1):130-8.

44. Hickey RJ, Zhou X, Pierson JD, Ravel J, Forney LJ. Understanding vaginal microbiome complexity from an ecological perspective. Transl Res. 2012;160(4):267-82.

45. Song SD, Acharya KD, Zhu JE, Deveney CM, Walther-Antonio MRS, Tetel MJ, et al. Daily Vaginal Microbiota Fluctuations Associated with Natural Hormonal Cycle, Contraceptives, Diet, and Exercise. mSphere. 2020;5(4).

46. Lebeer S, Ahannach S, Gehrmann T, Wittouck S, Eilers T, Oerlemans E, et al. A citizen-science-enabled catalogue of the vaginal microbiome and associated factors. Nat Microbiol. 2023;8(11):2183-95.

47. Krog MC, Hugerth LW, Fransson E, Bashir Z, Nyboe Andersen A, Edfeldt G, et al. The healthy female microbiome across body sites: effect of hormonal contraceptives and the menstrual cycle. Hum Reprod. 2022;37(7):1525-43.

48. Park MG, Cho S, Oh MM. Menopausal Changes in the Microbiome-A Review Focused on the Genitourinary Microbiome. Diagnostics (Basel). 2023;13(6).

49. Shen J, Song N, Williams CJ, Brown CJ, Yan Z, Xu C, et al. Effects of low dose estrogen therapy on the vaginal microbiomes of women with atrophic vaginitis. Sci Rep. 2016;6:24380.

50. Tomczyk K, Chmaj-Wierzchowska K, Wszołek K, Wilczak M. New Possibilities for Hormonal Vaginal Treatment in Menopausal Women. J Clin Med. 2023;12(14).

51. Hummelen R, Macklaim JM, Bisanz JE, Hammond JA, McMillan A, Vongsa R, et al. Vaginal microbiome and epithelial gene array in post-menopausal women with moderate to severe dryness. PLoS One. 2011;6(11):e26602.

52. Cinicola B, Conti MG, Terrin G, Sgrulletti M, Elfeky R, Carsetti R, et al. The Protective Role of Maternal Immunization in Early Life. Front Pediatr. 2021;9:638871.

53. Woods RM, Lorusso JM, Fletcher J, ElTaher H, McEwan F, Harris I, et al. Maternal immune activation and role of placenta in the prenatal programming of neurodevelopmental disorders. Neuronal Signal. 2023;7(2):Ns20220064.

54. Goswami TK, Singh M, Dhawan M, Mitra S, Emran TB, Rabaan AA, et al. Regulatory T cells (Tregs) and their therapeutic potential against autoimmune disorders - Advances and challenges. Hum Vaccin Immunother. 2022;18(1):2035117.

55. PrabhuDas M, Bonney E, Caron K, Dey S, Erlebacher A, Fazleabas A, et al. Immune mechanisms at the maternal-fetal interface: perspectives and challenges. Nat Immunol. 2015;16(4):328-34.

56. Zhang X, Wei H. Role of Decidual Natural Killer Cells in Human Pregnancy and Related Pregnancy Complications. Front Immunol. 2021;12:728291.

57. Walther-António MR, Jeraldo P, Berg Miller ME, Yeoman CJ, Nelson KE, Wilson BA, et al. Pregnancy's stronghold on the vaginal microbiome. PLoS One. 2014;9(6):e98514.

58. Aagaard K, Riehle K, Ma J, Segata N, Mistretta TA, Coarfa C, et al. A metagenomic approach to characterization of the vaginal microbiome signature in pregnancy. PLoS One. 2012;7(6):e36466.

59. Lehtoranta L, Ala-Jaakkola R, Laitila A, Maukonen J. Healthy Vaginal Microbiota and Influence of Probiotics Across the Female Life Span. Front Microbiol. 2022;13:819958.

60. Nelson TM, Borgogna JC, Michalek RD, Roberts DW, Rath JM, Glover ED, et al. Cigarette smoking is associated with an altered vaginal tract metabolomic profile. Scientific Reports. 2018;8(1):852.

61. Ponomarova I, Lisyana T, Matyashova O, Krishchuk S. The state of the microbiota of the genital tract in women who smoke. Medical Research Journal. 2023;8(2):147-51.

62. Lacroix G, Gouyer V, Gottrand F, Desseyn JL. The Cervicovaginal Mucus Barrier. Int J Mol Sci. 2020;21(21).

63. Amabebe E, Anumba DOC. Psychosocial Stress, Cortisol Levels, and Maintenance of Vaginal Health. Frontiers in Endocrinology. 2018;9.

64. Balle C, Konstantinus IN, Jaumdally SZ, Havyarimana E, Lennard K, Esra R, et al. Hormonal contraception alters vaginal microbiota and cytokines in South African adolescents in a randomized trial. Nat Commun. 2020;11(1):5578.

65. Brooks JP, Edwards DJ, Blithe DL, Fettweis JM, Serrano MG, Sheth NU, et al. Effects of combined oral contraceptives, depot medroxyprogesterone acetate and the levonorgestrel-releasing intrauterine system on the vaginal microbiome. Contraception. 2017;95(4):405-13.

66. Dabee S, Tanko RF, Brown BP, Bunjun R, Balle C, Feng C, et al. Comparison of Female Genital Tract Cytokine and Microbiota Signatures Induced by Initiation of Intramuscular DMPA and NET-EN Hormonal Contraceptives - a Prospective Cohort Analysis. Front Immunol. 2021;12:760504.

67. Wessels JM, Lajoie J, Cooper M, Omollo K, Felker AM, Vitali D, et al. Medroxyprogesterone acetate alters the vaginal microbiota and microenvironment in women and increases susceptibility to HIV-1 in humanized mice. Dis Model Mech. 2019;12(10).

68. Jacobson JC, Turok DK, Dermish AI, Nygaard IE, Settles ML. Vaginal microbiome changes with levonorgestrel intrauterine system placement. Contraception. 2014;90(2):130-5.

69. Donders G, Bellen G, Janssens D, Van Bulck B, Hinoul P, Verguts J. Influence of contraceptive choice on vaginal bacterial and fungal microflora. European Journal of Clinical Microbiology & Infectious Diseases. 2017;36(1):43-8.

70. Erol O, Simavlı S, Derbent AU, Ayrım A, Kafalı H. The impact of copper-containing and levonorgestrel-releasing intrauterine contraceptives on cervicovaginal cytology and microbiological flora: A prospective study. The European Journal of Contraception & Reproductive Health Care. 2014;19(3):187-93.

71. Liu P, Lu Y, Li R, Chen X. Use of probiotic lactobacilli in the treatment of vaginal infections: In vitro and in vivo investigations. Front Cell Infect Microbiol. 2023;13:1153894.

72. Dong M, Dong Y, Bai J, Li H, Ma X, Li B, et al. Interactions between microbiota and cervical epithelial, immune, and mucus barrier. Front Cell Infect Microbiol. 2023;13:1124591.

73. Baecher-Allan C, Kaskow BJ, Weiner HL. Multiple Sclerosis: Mechanisms and Immunotherapy. Neuron. 2018;97(4):742-68.

74. Peng H, He X, Wang Q. Immune checkpoint blockades in gynecological cancers: A review of clinical trials. Acta Obstet Gynecol Scand. 2022;101(9):941-51.

75. Lorscheider J, Benkert P, Lienert C, Hänni P, Derfuss T, Kuhle J, et al. Comparative analysis of dimethyl fumarate and fingolimod in relapsing-remitting multiple sclerosis. J Neurol. 2021;268(3):941-9.

76. Vališ M, Ryška P, Halúsková S, Klímová B, Pavelek Z. Highly active RRMS and ocrelizumab after failure of alemtuzumab therapy. BMC Neurol. 2020;20(1):202.

77. Canto-Gomes J, Boleixa D, Teixeira C, Martins da Silva A, González-Suárez I, Cerqueira J, et al. Distinct disease-modifying therapies are associated with different blood immune cell profiles in people with relapsing-remitting multiple sclerosis. International Immunopharmacology. 2024;131:111826.

78. Sabatino JJ, Jr., Zamvil SS, Hauser SL. B-Cell Therapies in Multiple Sclerosis. Cold Spring Harb Perspect Med. 2019;9(2).

79. Krumbholz M, Meinl I, Kümpfel T, Hohlfeld R, Meinl E. Natalizumab disproportionately increases circulating pre-B and B cells in multiple sclerosis. Neurology. 2008;71(17):1350-4.

80. Longbrake EE, Cross AH. Effect of Multiple Sclerosis Disease-Modifying Therapies on B Cells and Humoral Immunity. JAMA Neurol. 2016;73(2):219-25.

81. de Sèze J, Maillart E, Gueguen A, Laplaud DA, Michel L, Thouvenot E, et al. Anti-CD20 therapies in multiple sclerosis: From pathology to the clinic. Frontiers in Immunology. 2023;14.

82. Harris S, Feagan BG, Hanauer S, Vermeire S, Ghosh S, Yan J, et al. Ozanimod Differentially Impacts Circulating Lymphocyte Subsets in Patients with Moderately to Severely Active Crohn's Disease. Dig Dis Sci. 2024;69(6):2044-54.

83. Al-Nasiry S, Ambrosino E, Schlaepfer M, Morré SA, Wieten L, Voncken JW, et al. The Interplay Between Reproductive Tract Microbiota and Immunological System in Human Reproduction. Front Immunol. 2020;11:378.

84. Bresciani G, Manai F, Davinelli S, Tucci P, Saso L, Amadio M. Novel potential pharmacological applications of dimethyl fumarate—an overview and update. Frontiers in Pharmacology. 2023;14.

85. Pang X, He X, Qiu Z, Zhang H, Xie R, Liu Z, et al. Targeting integrin pathways: mechanisms and advances in therapy. Signal Transduction and Targeted Therapy. 2023;8(1):1.

86. Ma LL, Wu ZT, Wang L, Zhang XF, Wang J, Chen C, et al. Inhibition of hepatic cytochrome P450 enzymes and sodium/bile acid cotransporter exacerbates leflunomide-induced hepatotoxicity. Acta Pharmacol Sin. 2016;37(3):415-24.

87. Mhanna E, Nouchi A, Louapre C, De Paz R, Heinzlef O, Bodini B, et al. Human papillomavirus lesions in 16 MS patients treated with fingolimod: Outcomes and vaccination. Multiple Sclerosis Journal. 2021;27(11):1794-8.

88. Macaron G, Ontaneda D. Clinical commentary on “Warts and all: Fingolimod and unusual HPV associated lesions”. Multiple Sclerosis Journal. 2019;25(11):1550-2.

89. Triplett J, Kermode AG, Corbett A, Reddel SW. Warts and all: Fingolimod and unusual HPV-associated lesions. Mult Scler. 2019;25(11):1547-50.

90. Paybast S, Ashtari F, Moghaddam NB, Poursadeghfard M, Abutorabi M, Nahayati MA, et al. Investigating treatment alternatives for fingolimod in patients with multiple sclerosis developed refractory fingolimod-related genital Human Papilloma Virus (HPV) infection. Multiple Sclerosis and Related Disorders. 2025;95:106284.

91. Stamatellos VP, Siafis S, Papazisis G. Disease-modifying agents for multiple sclerosis and the risk for reporting cancer: A disproportionality analysis using the US Food and Drug Administration Adverse Event Reporting System database. Br J Clin Pharmacol. 2021;87(12):4769-79.

92. Gaindh D, Kavak KS, Teter B, Vaughn CB, Cookfair D, Hahn T, et al. Decreased risk of cancer in multiple sclerosis patients and analysis of the effect of disease modifying therapies on cancer risk. J Neurol Sci. 2016;370:13-7.

93. Mariottini A, Forci B, Gualdani E, Romoli M, Repice AM, Barilaro A, et al. Incidence of malignant neoplasms and mortality in people affected by multiple sclerosis in the epoch of disease-modifying treatments: A population-based study on Tuscan residents. Mult Scler Relat Disord. 2022;60:103679.

94. Achiron A, Barak Y, Gail M, Mandel M, Pee D, Ayyagari R, et al. Cancer incidence in multiple sclerosis and effects of immunomodulatory treatments. Breast Cancer Res Treat. 2005;89(3):265-70.

95. Lebrun C, Vermersch P, Brassat D, Defer G, Rumbach L, Clavelou P, et al. Cancer and multiple sclerosis in the era of disease-modifying treatments. J Neurol. 2011;258(7):1304-11.

96. Dolladille C, Chrétien B, Peyro-Saint-Paul L, Alexandre J, Dejardin O, Fedrizzi S, et al. Association Between Disease-Modifying Therapies Prescribed to Persons with Multiple Sclerosis and Cancer: a WHO Pharmacovigilance Database Analysis. Neurotherapeutics. 2021;18(3):1657-64.

97. Gil-Bernal R, González-Caballero JL, Espinosa-Rosso R, Gómez-Gómez C. Potential risk of disease modifying therapies on neoplasm development and coadjutant factors in multiple sclerosis outpatients. Sci Rep. 2021;11(1):12533.

98. Ross L, Ng HS, O'Mahony J, Amato MP, Cohen JA, Harnegie MP, et al. Women's Health in Multiple Sclerosis: A Scoping Review. Front Neurol. 2021;12:812147.

99. Dobos K, Healy B, Houtchens M. Access to Preventive Health Care in Severely Disabled Women with Multiple Sclerosis. Int J MS Care. 2015;17(4):200-5.

100. Cheng E, Myers L, Wolf S, Shatin D, Cui XP, Ellison G, et al. Mobility impairments and use of preventive services in women with multiple sclerosis: observational study. Bmj. 2001;323(7319):968-9.

101. Papeix C, Donze C, Lebrun-Frénay C. Infections and multiple sclerosis: Recommendations from the French Multiple Sclerosis Society. Rev Neurol (Paris). 2021;177(8):980-94.

102. Iezzoni LI, Kurtz SG, Rao SR. Trends in Pap Testing Over Time for Women With and Without Chronic Disability. Am J Prev Med. 2016;50(2):210-9.

103. Horner-Johnson W, Dobbertin K, Andresen EM, Iezzoni LI. Breast and cervical cancer screening disparities associated with disability severity. Womens Health Issues. 2014;24(1):e147-53.

104. Andresen EM, Peterson-Besse JJ, Krahn GL, Walsh ES, Horner-Johnson W, Iezzoni LI. Pap, mammography, and clinical breast examination screening among women with disabilities: a systematic review. Womens Health Issues. 2013;23(4):e205-14.

105. Grytten N, Myhr KM, Celius EG, Benjaminsen E, Kampman MT, Midgard R, et al. Incidence of cancer in multiple sclerosis before and after the treatment era- a registry- based cohort study. Mult Scler Relat Disord. 2021;55:103209.

106. Lebrun C, Rocher F. Cancer Risk in Patients with Multiple Sclerosis: Potential Impact of Disease-Modifying Drugs. CNS Drugs. 2018;32(10):939-49.

107. Wan KM, Oehler MK. Rapid Progression of Low-Grade Cervical Dysplasia into Invasive Cancer during Natalizumab Treatment for Relapsing Remitting Multiple Sclerosis. Case Rep Oncol. 2019;12(1):59-62.

108. Rolfes L, Lokhorst B, Samijn J, van Puijenbroek E. Cervical dysplasia associated with the use of natalizumab. Neth J Med. 2013;71(9):494-5.

109. Durrieu G, Dardonville Q, Clanet M, Montastruc JL. Cervical dysplasia in a patient with multiple sclerosis treated with natalizumab. Fundam Clin Pharmacol. 2019;33(1):125-6.

110. Alping P, Askling J, Burman J, Fink K, Fogdell-Hahn A, Gunnarsson M, et al. Cancer Risk for Fingolimod, Natalizumab, and Rituximab in Multiple Sclerosis Patients. Ann Neurol. 2020;87(5):688-99.

111. Polman CH, O'Connor PW, Havrdova E, Hutchinson M, Kappos L, Miller DH, et al. A randomized, placebo-controlled trial of natalizumab for relapsing multiple sclerosis. N Engl J Med. 2006;354(9):899-910.

112. Kappos L, Polman CH, Freedman MS, Edan G, Hartung HP, Miller DH, et al. Treatment with interferon beta-1b delays conversion to clinically definite and McDonald MS in patients with clinically isolated syndromes. Neurology. 2006;67(7):1242-9.

113. Wolinsky JS, Borresen TE, Dietrich DW, Wynn D, Sidi Y, Steinerman JR, et al. GLACIER: An open-label, randomized, multicenter study to assess the safety and tolerability of glatiramer acetate 40 mg three-times weekly versus 20 mg daily in patients with relapsing-remitting multiple sclerosis. Mult Scler Relat Disord. 2015;4(4):370-6.

114. Reder AT, Ebers GC, Traboulsee A, Li D, Langdon D, Goodin DS, et al. Cross-sectional study assessing long-term safety of interferon-beta-1b for relapsing-remitting MS. Neurology. 2010;74(23):1877-85.

115. O'Connor P, Filippi M, Arnason B, Comi G, Cook S, Goodin D, et al. 250 microg or 500 microg interferon beta-1b versus 20 mg glatiramer acetate in relapsing-remitting multiple sclerosis: a prospective, randomised, multicentre study. Lancet Neurol. 2009;8(10):889-97.

116. Doosti R, Togha M, Moghadasi AN, Aghsaie A, Azimi AR, Khorramnia S, et al. Evaluation of the risk of cervical cancer in patients with Multiple Sclerosis treated with cytotoxic agents: A cohort study. Iran J Neurol. 2018;17(2):64-70.

117. Bridge F, Brotherton J, Stankovich J, Sanfilippo PG, Skibina OG, Buzzard K, et al. Risk of Cervical Abnormalities for Women With Multiple Sclerosis Treated With Moderate-Efficacy and High-Efficacy Disease-Modifying Therapies. Neurology. 2024;102(4):e208059.

118. Reusser NM, Downing C, Guidry J, Tyring SK. HPV Carcinomas in Immunocompromised Patients. J Clin Med. 2015;4(2):260-81.

119. Grulich AE, van Leeuwen MT, Falster MO, Vajdic CM. Incidence of cancers in people with HIV/AIDS compared with immunosuppressed transplant recipients: a meta-analysis. Lancet. 2007;370(9581):59-67.

120. Markowitz LE, Tsu V, Deeks SL, Cubie H, Wang SA, Vicari AS, et al. Human papillomavirus vaccine introduction--the first five years. Vaccine. 2012;30 Suppl 5:F139-48.

121. Moscicki A-B, Flowers L, Huchko MJ, Long ME, MacLaughlin KL, Murphy J, et al. Updated Review for Guidelines for Cervical Cancer Screening in Immunosuppressed Women Without HIV Infection. Journal of Lower Genital Tract Disease. 9900.

122. Leung SOA, Akinwunmi B, Elias KM, Feldman S. Educating healthcare providers to increase Human Papillomavirus (HPV) vaccination rates: A Qualitative Systematic Review. Vaccine: X. 2019;3:100037.

123. El-Zein M, Richardson L, Franco EL. Cervical cancer screening of HPV vaccinated populations: Cytology, molecular testing, both or none. J Clin Virol. 2016;76 Suppl 1(Suppl 1):S62-s8.

124. Pathak P, Pajai S, Kesharwani H. A Review on the Use of the HPV Vaccine in the Prevention of Cervical Cancer. Cureus. 2022;14(9):e28710.

125. Clifford GM, Gonçalves MA, Franceschi S. Human papillomavirus types among women infected with HIV: a meta-analysis. Aids. 2006;20(18):2337-44.

126. Garland SM, Brotherton JML, Moscicki AB, Kaufmann AM, Stanley M, Bhatla N, et al. HPV vaccination of immunocompromised hosts. Papillomavirus Res. 2017;4:35-8.

127. Prevention CfDCa. Human papillomavirus (HPV) and cancer 2021 [Available from: <https://www.cdc.gov/std/treatment-guidelines/hpv-cancer.htm>.

128. Moscicki A-B, Flowers L, Huchko MJ, Long ME, MacLaughlin KL, Murphy J, et al. Guidelines for Cervical Cancer Screening in Immunosuppressed Women Without HIV Infection. Journal of Lower Genital Tract Disease. 2019;23(2).

129. Perkins RB, Guido RS, Castle PE, Chelmow D, Einstein MH, Garcia F, et al. 2019 ASCCP Risk-Based Management Consensus Guidelines for Abnormal Cervical Cancer Screening Tests and Cancer Precursors. J Low Genit Tract Dis. 2020;24(2):102-31.

130. Sotzen JR, Stratman EJ. Vulvovaginal pyoderma gangrenosum associated with rituximab use in 2 patients with rheumatoid arthritis. JAAD Case Reports. 2021;10:75-7.

131. Walsh M, Leonard N, Bell H. Superficial Granulomatous Pyoderma of the Vulva in a Patient Receiving Maintenance Rituximab (MabThera) for Lymphoma. Journal of Lower Genital Tract Disease. 2011;15(2).

132. Georgakopoulos JR, Rohekar G, Lovegrove FE. A case of rituximab-induced pyoderma gangrenosum. JAAD Case Reports. 2018;4(10):979-81.

133. Vikse J, Rygh A, Kaisen K, Omdal R. Life-threatening rituximab-induced pyoderma gangrenosum successfully treated with intravenous immunoglobulin. Scandinavian Journal of Rheumatology. 2017;46(5):413-4.

134. Selva-Nayagam P, Fischer G, Hamann I, Sobel J, James C. Rituximab Causing Deep Ulcerative Suppurative Vaginitis/Pyoderma Gangrenosum. Current Infectious Disease Reports. 2015;17(5):23.

135. Maloney C, Blickenstaff N, Kugasia A, Buford LB, Hoffman MD. Vulvovaginal pyoderma gangrenosum in association with rituximab. JAAD Case Reports. 2018;4(9):907-9.

136. Selva-Nayagam P, Fischer G, Hamann I, Sobel J, James C. Rituximab causing deep ulcerative suppurative vaginitis/pyoderma gangrenosum. Curr Infect Dis Rep. 2015;17(5):478.

137. Wang JY, French LE, Shear NH, Amiri A, Alavi A. Drug-Induced Pyoderma Gangrenosum: A Review. American Journal of Clinical Dermatology. 2018;19(1):67-77.

138. Walsh M, Leonard N, Bell H. Superficial granulomatous pyoderma of the vulva in a patient receiving maintenance rituximab (MabThera) for lymphoma. J Low Genit Tract Dis. 2011;15(2):158-60.

139. Vikse J, Rygh A, Kaisen K, Omdal R. Life-threatening rituximab-induced pyoderma gangrenosum successfully treated with intravenous immunoglobulin. Scand J Rheumatol. 2017;46(5):413-4.

140. Dixit S, Selva-Nayagam P, Hamann I, Fischer G. Vulvovaginal pyoderma gangrenosum secondary to rituximab therapy. J Low Genit Tract Dis. 2015;19(1):e6-9.

141. Georgakopoulos JR, Rohekar G, Lovegrove FE. A case of rituximab-induced pyoderma gangrenosum. JAAD Case Rep. 2018;4(10):979-81.

142. Bell AP, Jr., Custer MK, Presley S. Pyoderma Gangrenosum in a 75-Year-Old Male With Follicular Diffuse Large B-cell Lymphoma. Cureus. 2024;16(8):e67234.

143. Yockey L, Dowst S, Zonozi R, Huizenga N, Murphy P, Laliberte K, et al. Inflammatory vaginitis in women on long-term rituximab treatment for autoimmune disorders. BMC Women's Health. 2021;21(1):285.

144. Klumpp A, Luessi F, Engel S, Weidenthaler-Barth B, Becker D, Grabbe S, et al. Ocrelizumab-induced vulvovaginal pyoderma gangrenosum in a patient with relapsing-remitting multiple sclerosis. JAAD Case Rep. 2022;28:24-7.

145. Parrotta E, Kopinsky H, Abate J, Ryerson LZ, Krupp LB. It's not always an infection: Pyoderma gangrenosum of the urogenital tract in two patients with multiple sclerosis treated with rituximab. Multiple Sclerosis and Related Disorders. 2023;70.

146. Breneman AN, Eber AE, Haque H, Levine L, Askanase A, Riley CS, et al. Vulvovaginal Pyoderma Gangrenosum in a Patient Treated With Ocrelizumab for Multiple Sclerosis. J Low Genit Tract Dis. 2022;26(2):189-91.

147. Conway S, Dodson C, Hui G, William Pike C, Galetta K. Inflammatory vaginitis in women with multiple sclerosis: a retrospective analysis of B-cell depleting therapy compared to other disease modifying therapies. Mult Scler Relat Disord. 2024;92:105921.

148. Makris G-M, Mene J, Fotiou A, Xyla V, Battista M-J, Sergentanis TN. Gynecological adverse effects of natalizumab administration: Case report and review of the literature. Multiple Sclerosis and Related Disorders. 2018;25:46-9.

149. Levine L, Son J, Yu A, Wesley S, De Jager PL, Moynihan E, et al. Inflammatory vaginitis in four B-cell suppressed women with Multiple Sclerosis. Multiple Sclerosis and Related Disorders. 2024;82.

150. Chen J, He R, Sun W, Gao R, Peng Q, Zhu L, et al. TAGAP instructs Th17 differentiation by bridging Dectin activation to EPHB2 signaling in innate antifungal response. Nat Commun. 2020;11(1):1913.

151. Sun W, Ma X, Wang H, Du Y, Chen J, Hu H, et al. MYO1F regulates antifungal immunity by regulating acetylation of microtubules. Proc Natl Acad Sci U S A. 2021;118(30).

152. Saroukolaei SA, Ghabaee M, Shokri H, Badiei A, Ghourchian S. The role of Candida albicans in the severity of multiple sclerosis. Mycoses. 2016;59(11):697-704.

153. Benito-León J, Pisa D, Alonso R, Calleja P, Díaz-Sánchez M, Carrasco L. Association between multiple sclerosis and Candida species: evidence from a case-control study. Eur J Clin Microbiol Infect Dis. 2010;29(9):1139-45.

154. Pisa D, Alonso R, Jiménez-Jiménez FJ, Carrasco L. Fungal infection in cerebrospinal fluid from some patients with multiple sclerosis. European Journal of Clinical Microbiology & Infectious Diseases. 2013;32(6):795-801.

155. Hollenbach JA, Oksenberg JR. The immunogenetics of multiple sclerosis: A comprehensive review. Journal of Autoimmunity. 2015;64:13-25.

156. Benito-León J, Laurence M. The Role of Fungi in the Etiology of Multiple Sclerosis. Frontiers in Neurology. 2017;8.

157. Fraga-Silva TF, Mimura LA, Marchetti CM, Chiuso-Minicucci F, França TG, Zorzella-Pezavento SF, et al. Experimental autoimmune encephalomyelitis development is aggravated by Candida albicans infection. J Immunol Res. 2015;2015:635052.

158. Yadav M, Ali S, Shrode RL, Shahi SK, Jensen SN, Hoang J, et al. Multiple sclerosis patients have an altered gut mycobiome and increased fungal to bacterial richness. PLoS One. 2022;17(4):e0264556.

159. Ram R, Ben-Bassat I, Shpilberg O, Polliack A, Raanani P. The late adverse events of rituximab therapy--rare but there! Leuk Lymphoma. 2009;50(7):1083-95.

160. Aggarwal P. Pyoderma gangrenosum adverse event with Rituximab use: A postmarketing pharmacovigilance analysis. Dermatol Ther. 2020;33(2):e13221.

161. Frobenius W, Bogdan C. Diagnostic Value of Vaginal Discharge, Wet Mount and Vaginal pH - An Update on the Basics of Gynecologic Infectiology. Geburtshilfe Frauenheilkd. 2015;75(4):355-66.

162. Coles AJ, Twyman CL, Arnold DL, Cohen JA, Confavreux C, Fox EJ, et al. Alemtuzumab for patients with relapsing multiple sclerosis after disease-modifying therapy: a randomised controlled phase 3 trial. The lancet. 2012;380(9856):1829-39.

163. Cohen JA, Coles AJ, Arnold DL, Confavreux C, Fox EJ, Hartung H-P, et al. Alemtuzumab versus interferon beta 1a as first-line treatment for patients with relapsing-remitting multiple sclerosis: a randomised controlled phase 3 trial. The Lancet. 2012;380(9856):1819-28.

164. Cohen JA, Barkhof F, Comi G, Hartung H-P, Khatri BO, Montalban X, et al. Oral fingolimod or intramuscular interferon for relapsing multiple sclerosis. New England Journal of Medicine. 2010;362(5):402-15.

165. Arvin AM, Wolinsky JS, Kappos L, Morris MI, Reder AT, Tornatore C, et al. Varicella-zoster virus infections in patients treated with fingolimod: risk assessment and consensus recommendations for management. JAMA neurology. 2015;72(1):31-9.

166. Montalban X, Hauser SL, Kappos L, Arnold DL, Bar-Or A, Comi G, et al. Ocrelizumab versus placebo in primary progressive multiple sclerosis. New england journal of medicine. 2017;376(3):209-20.

167. Hauser SL, Bar-Or A, Comi G, Giovannoni G, Hartung H-P, Hemmer B, et al. Ocrelizumab versus interferon beta-1a in relapsing multiple sclerosis. New England Journal of Medicine. 2017;376(3):221-34.

168. Ma BB, Ostrow LW, Newsome SD. Disseminated zoster with paresis in a multiple sclerosis patient treated with dimethyl fumarate. Neuroimmunology & Neuroinflammation. 2016;3(2):e203.

169. (CDC) CfDCaP. Genital Herpes – CDC Treatment Guidelines. 2025.

170. McCauley KE, Rackaityte E, LaMere B, Fadrosh DW, Fujimura KE, Panzer AR, et al. Heritable vaginal bacteria influence immune tolerance and relate to early-life markers of allergic sensitization in infancy. Cell Rep Med. 2022;3(8):100713.

171. Gilbert JA, Quinn RA, Debelius J, Xu ZZ, Morton J, Garg N, et al. Microbiome-wide association studies link dynamic microbial consortia to disease. Nature. 2016;535(7610):94-103.

72. Nyangahu DD, Jaspan HB. Influence of maternal microbiota during pregnancy on infant immunity. Clin Exp Immunol. 2019;198(1):47-56.

173. Sereme Y, Toumi E, Saifi E, Faury H, Skurnik D. Maternal immune factors involved in the prevention or facilitation of neonatal bacterial infections. Cellular Immunology. 2024;395-396:104796.

174. Centers for Disease Control and Prevention. Human Papillomavirus (HPV) Vaccination: Recommendations of the Advisory Committee on Immunization Practices (ACIP). Updated February 8 AD, 2024. .
